# Effect of plasma polyunsaturated fatty acid levels on leukocyte telomere lengths in the Singaporean Chinese population

**DOI:** 10.1186/s12937-020-00626-9

**Published:** 2020-10-30

**Authors:** Xuling Chang, Rajkumar Dorajoo, Ye Sun, Ling Wang, Choon Nam Ong, Jianjun Liu, Chiea Chuen Khor, Jian-Min Yuan, Woon Puay Koh, Yechiel Friedlander, Chew-Kiat Heng

**Affiliations:** 1 Department of Paediatrics, Yong Loo Lin School of Medicine, National University of Singapore, NUHS Tower Block, Level 12, 1E Kent Ridge Road, Singapore, 119228 Singapore; 2 Khoo Teck Puat - National University Children’s Medical Institute, National University Health System, Singapore, Singapore; 3 Genome Institute of Singapore, Agency for Science, Technology and Research, Singapore, Singapore; 4 Nestlé Research Singapore Hub, Singapore, 21 Biopolis Drive, Nucleos, Singapore, Singapore; 5 Saw Swee Hock School of Public Health, National University of Singapore, National University Health System, Singapore, Singapore; 6 NUS Environmental Research Institute, National University of Singapore, Singapore, Singapore; 7 Department of Medicine, Yong Loo Lin School of Medicine, National University of Singapore, Singapore, Singapore; 8 Singapore Eye Research Institute, Singapore National Eye Centre, Singapore, Singapore; 9 Division of Cancer Control and Population Sciences, UPMC Hillman Cancer Center, University of Pittsburgh, Pittsburgh, PA USA; 10 Department of Epidemiology, Graduate School of Public Health, University of Pittsburgh, Pittsburgh, PA USA; 11 Health Systems and Services Research, Duke-NUS Medical School Singapore, Singapore, Singapore; 12 School of Public Health and Community Medicine, Hebrew University of Jerusalem, Jerusalem, Israel; 13 Unit of Epidemiology, Hebrew University-Hadassah Braun School of Public Health, POB 12272, 91120 Jerusalem, Israel

**Keywords:** Polyunsaturated fatty acid, Leukocyte telomere length, Gene-diet interaction

## Abstract

**Background:**

Shorter telomere length (TL) has been associated with poor health behaviors, increased risks of chronic diseases and early mortality. Excessive shortening of telomere is a marker of accelerated aging and can be influenced by oxidative stress and nutritional deficiency. Plasma n6:n3 polyunsaturated fatty acid (PUFA) ratio may impact cell aging. Increased dietary intake of marine n-3 PUFA is associated with reduced telomere attrition. However, the effect of plasma PUFA on leukocyte telomere length (LTL) and its interaction with genetic variants are not well established.

**Methods:**

A nested coronary artery disease (CAD) case-control study comprising 711 cases and 638 controls was conducted within the Singapore Chinese Health Study (SCHS). Samples genotyped with the Illumina ZhongHua-8 array. Plasma n-3 and n-6 PUFA were quantified using mass spectrometry (MS). LTL was measured with quantitative PCR method. Linear regression was used to test the association between PUFA and LTL. The interaction between plasma PUFAs and genetic variants was assessed by introducing an additional term (PUFA×genetic variant) in the regression model. Analysis was carried out in cases and controls separately and subsequently meta-analyzed using the inverse-variance weighted method. We further assessed the association of PUFA and LTL with CAD risk by Cox Proportional-Hazards model and whether the effect of PUFA on CAD was mediated through LTL by using structural equation modeling.

**Results:**

Higher n6:n3 ratio was significantly associated with shorter LTL (*p* = 0.018) and increased CAD risk (*p* = 0.005). These associations were mainly driven by elevated plasma total n-3 PUFAs, especially eicosapentaenoic acid (EPA) and docosahexaenoic acid (DHA) (*p* < 0.05). There was a statistically significant interaction for an intergenic single nucleotide polymorphism (SNP) rs529143 with plasma total n-3 PUFA and DHA on LTL beyond the genome-wide threshold (*p* < 5 ×  10^− 8^). Mediation analysis showed that PUFA and LTL affected CAD risk independently.

**Conclusions:**

Higher plasma n6:n3 PUFA ratio, and lower EPA and DHA n-3 PUFAs were associated with shorter LTL and increased CAD risk in this Chinese population. Furthermore, genetic variants may modify the effect of PUFAs on LTL. PUFA and LTL had independent effect on CAD risk in our study population.

## Introduction

Telomeres are complexes at the ends of eukaryotic chromosomes, which consist of tandem repeat DNA sequences (TTAGGG)_n_ for humans, and associated proteins [1]. Telomeres protects the genome from degradation, interchromosomal fusion, unnecessary recombination and being recognized as a double-strand break by DNA repair proteins [2]. Telomeres shorten progressively during cell divisions and eventually result in cellular senescence or apoptosis when telomere length (TL) reaches a critical limit [3]. Growing epidemiologic and clinical studies have shown that TL is associated with chronic diseases, including cancer [4], osteoporosis [5], and cardiovascular diseases [6]. These observations have led to telomeres being proposed as an important marker of biological age, which is independent of chronological age [7], and as a prognostic marker of chronic disease risk, progression and premature mortality [8, 9].

Dietary intake is a significant contributor in determining cellular TL. Intakes of both omega 3 (n-3) and omega-6 (n-6) polyunsaturated fatty acids (PUFAs) can influence inflammation [10], which may affect telomeres attrition rate both in vitro [11] and in vivo [12]. Higher n-3 PUFA concentration in plasma may have an anti-inflammatory effect [13], while n-6 PUFA shows pro-inflammatory and pro-thrombotic potential through synthesis of oxidized metabolites [14, 15]. There is competition between n-3 and n-6 PUFA for desaturation and elongation enzymes. The ratio of plasma n-6 and n-3 PUFA (n6:n3 ratio), may hence contribute to inflammatory profiles and health status of an individual [16].

Genetic studies have identified several loci associated with leukocyte telomere length (LTL) [17, 18]. However, the contribution of these variants, even in combination, to the overall heritability of LTL is modest. Interaction between genes and life-style factors may also contribute to LTL heritability. The aims of this study were to investigate the association between plasma PUFA levels and LTL in the Chinese population and to evaluate genetic variants that may modify this effect.

## Method

### Study population

The Singapore Chinese Health Study (SCHS) is a long-term population-based prospective cohort study focused on dietary, genetic and environmental determinants of cancer and other chronic diseases in Singapore [19]. From April 1993 to December 1998, a total of 63,257 Chinese individuals (Hokkien or Cantonese dialect group) aged 45–75 years were recruited. At recruitment, all the study subjects were interviewed in-person by an interviewer with a structured questionnaire. Since April 1994, a total of 28,439 participants donated blood specimens. The study was approved by the Institutional Review Boards of the National University of Singapore and the University of Minnesota, and all study subjects gave written informed consent.

The current study was conducted in a coronary artery diseases (CAD) case-control study nested within SCHS, including 744 incident acute myocardial infarction (AMI) cases and 744 matched controls. Both cases and controls were SCHS participants with donated blood specimens and without a prior history of CAD or stroke at the time of blood collection. The cases selected were incident nonfatal or fatal AMI that occurred during follow-up from blood drawn through December 31, 2010. The controls were alive and free of CAD at the time of the AMI diagnosis or death of the index case. The matching criteria included gender, dialect group (Hokkien, Cantonese), date of birth (±5 years), date of recruitment (±2.5 years), and date of blood collection (±6 months) [20].

### Measurement of leukocyte telomere length

DNA of SCHS study subjects was extracted from peripheral blood collected prior to CAD events, using QIAamp DNA Blood kits (Qiagen, Valencia, CA). Relative LTL was measured using a validated monochrome multiplex quantitative PCR (qPCR) method [21]. This method expressed LTL as a ratio (T/S) of telomere repeat length (T) to copy number of a single copy gene albumin (S), relative to a reference sample. The LTL for each sample was measured in duplicates and the average T/S ratio was used for subsequent analysis. Detailed description for LTL measurement in SCHS, including standard curve generation, PCR condition and coefficients of variation was published previously [18].

### Measurement of plasma PUFA

Plasma n-3 and n-6 PUFA were quantified from baseline specimens prior to CAD events, in a targeted mode using gas chromatography–mass spectrometry (GC-MS)/MS on an Agilent 7890 GC system (Shanghai, China) equipped with a G7000B QQQ triple quadrupole mass detector and an auto sample injector. Both free and esterified (triglycerides, phospholipids, cholesterol esters) FA fractions were measured in total. Samples were analyzed in 76 batches, with cases and matched controls included in the same batch. Pooled human plasma was used for quality control (QC). The experimental details and the coefficients of variation of the measured FAs were published elsewhere [20].

### Genotyping and imputation

Study samples were genotyped on the Illumina HumanOmni ZhongHua-8 Bead Chip. After QC [20, 22–24] procedures, 711 cases and 638 controls with complete information for both genotypes and plasma PUFA measurement were included in the current study. Imputation for additional autosomal single nucleotide polymorphisms (SNPs) was performed with IMPUTE2 [25] and genotype calls were based on phase3 1000G cosmopolitan panels.

### Statistical method

The main demographic clinical characteristics for the study subjects were compared between CAD cases and controls. Normally distributed quantitative traits, including age, LTL, total plasma n-6 PUFA, linoleic acid (LA) and arachidonic acid (AA), were presented as mean ± SD (standard deviation) and the differences in means between cases and controls were compared by t-test. Non-normally distributed variables, including n6:n3 ratio, total plasma n-3 PUFA, α-linolenic acid (ALA), eicosapentaenoic acid (EPA), docosahexaenoic acid (DHA), γ-linolenic acid (GLA) and dihomo-γ-linolenic acid (DGLA) were presented as median with interquartile range, and the differences between groups were determined by the Mann-Whitney U test. Categorical variables, including gender and SNP genotypes, were presented as number of individuals and differences in their frequencies between groups were determined by Pearson’s χ^2^ test, which was also used for checking significant departure of genotype frequencies from Hardy–Weinberg expectations (HWE). Linear regression was used to investigate the main association of LTL with plasma PUFA and SNP. Cox Proportional-Hazards model was utilized to assess the association of PUFA and LTL with CAD risk, with age, gender and the first three principle components (PCs) included as covariates. Mediation analysis was conducted using the Structural Equation Modeling (SEM) to assess whether the effect of plasma PUFA on CAD risk was mediated through LTL. Non-normally distributed variables were normalized by z-score transformation. Genome-wide interaction analyses were also performed using linear regression by additionally introducing the interaction term (plasma PUFA x SNP) with PUFA and SNP included as covariates in the same regression model. Analysis was first carried out in cases and controls separately and subsequently meta-analyzed using the fixed-effects inverse-variance weighted method. Cochran’s Q test was used to measure heterogeneity and a Q_p_ value cut-off < 0.05 was used to determine SNPs with between-study heterogeneity [26]. The genome-wide interaction analysis was carried out by using an additive model in ProbABEL [27]. Common SNPs with minor allele frequency (MAF) above 3% were included in the current study. All other statistical analyses were carried out using STATA 15.0 (Stata Corp, College station, TX) and a 5% type I error was set to indicate statistical significance (two-tailed) in all analyses.

## Results

The main demographic characteristics for the study subjects were presented in Table 1. Cases had significantly lower plasma total n-3 PUFA levels (*p* = 0.013), EPA levels (*p* = 0.002) and DHA levels (*p* = 0.020) as compared to controls. No significant difference was observed between cases and controls for age, gender, LTL, plasma n6:n3 ratio, ALA, total n-6 PUFA and PUFA subtypes.

**Table 1:** Clinical characteristics of the study subjects

	CAD cases	CAD controls	*P*
	*N* = 711	*N* = 638	
Age (years)	66.64 ± 7.84	66.37 ± 7.82	0.531
Telomere length (T/S ratio)	1.00 ± 0.23	1.02 ± 0.24	0.100
Plasma fatty acid (% of total)			
n-3 fatty acid (%)	2.63 (2.04, 3.69)	2.83 (2.11, 4.02)	**0.013**
18:3 (n-3) ALA (%)	0.28 (0.21, 0.38)	0.28 (0.22, 0.38)	0.310
20:5 (n-3) EPA (%)	0.39 (0.31, 0.49)	0.42 (0.32, 0.53)	**0.002**
22:6 (n-3) DHA (%)	1.92 (1.38, 2.79)	2.02 (1.45, 3.05)	**0.020**
n-6 fatty acid (%)	45.07 ± 4.68	45.43 ± 4.81	0.165
18:2(n-6) LA (%)	36.14 ± 4.63	36.43 ± 4.68	0.252
18:3(n-6) GLA (%)	0.19 (0.13, 0.29)	0.19 (0.13, 0.29)	0.824
20:3(n-6) DGLA (%)	0.88 (0.67, 1.13)	0.87 (0.69, 1.10)	0.652
20:4(n-6) AA (%)	7.57 ± 1.68	7.67 ± 1.79	0.328
n6:n3 ratio	17.02 (11.98, 22.10)	16.14 (10.93, 21.62)	0.053
Gender (%male)	459 (64.56%)	401 (62.85%)	0.516
rs529143			0.759
AA	522 (75.98%)	461 (74.47%)	
AC	155 (22.56%)	150 (24.23%)	
CC	10 (1.46%)	8 (1.29%)	
MAF	0.127	0.134	

Data was presented as Mean ± Standard Deviation (SD) for normally distributed variables, median (interquartile range) for non-normally distributed variables or N (%) for categorical variables

*ALA* α-Linolenic acid; *EPA* Eicosapentaenoic acid; *DHA* Docosahexaenoic acid; *LA* Linoleic acid; *GLA* γ-Linolenic acid; *DGLA* Dihomo-γ-linolenic acid; *AA* Arachidonic acid; *MAF* Minor allele frequency

### Association between plasma PUFA and LTL

Higher plasma n6:n3 ratio was significantly associated with shorter LTL (T/S ratio) (β = − 0.015, *p* = 0.018, Table 2). When analyzing the individual effect of plasma n-3 and n-6 PUFA on LTL, only n-3 PUFA showed a significant association and each 1-SD increase in total n-3 PUFA was associated with 0.014 increase in relative LTL (β = 0.014, SE = 0.006, *p* = 0.024). We further analyzed the association between specific n-3 and n-6 PUFA subtypes with LTL. Both EPA and DHA showed significant associations with LTL while ALA did not. Each 1-SD increase of EPA and DHA was associated with 0.016 (β = 0.016, SE = 0.006, *p* = 0.011) and 0.015 (β = 0.015, SE = 0.006, *p* = 0.017) longer relative LTL, respectively (Table 2). The n-6 PUFA subtypes were not associated with LTL (Table 2).

**Table 2:** Association between plasma PUFA and telomere length in the individual datasets and after meta-analysis

	SCHS_CAD cases			SCHS_CAD control			Meta-analysis			
	*N* = 711			*N* = 638			*N* = 1349			
	beta	se	*p*	beta	se	*p*	beta	se	*p*	Q_p-value_
n6:n3 ratio	−0.008	0.009	0.362	−0.024	0.009	0.011	−0.015	0.006	0.018	0.202
n-3 fatty acid	0.005	0.009	0.535	0.025	0.009	0.007	0.014	0.006	0.024	0.114
18:3 (n-3) ALA	0.010	0.009	0.251	−0.008	0.009	0.403	0.002	0.006	0.769	0.164
20:5 (n-3) EPA	0.010	0.009	0.240	0.023	0.009	0.014	0.016	0.006	0.011	0.294
22:6 (n-3) DHA	0.004	0.009	0.622	0.029	0.009	0.003	0.015	0.006	0.017	0.054
n-6 fatty acid	−0.002	0.002	0.260	4.92× 10^−4^	0.002	0.806	−0.001	0.001	0.503	0.348
18:2(n-6) LA	−0.002	0.002	0.408	1.47× 10^−4^	0.002	0.943	−0.001	0.001	0.570	0.544
18:3(n-6) GLA	0.012	0.009	0.187	0.003	0.010	0.772	0.008	0.007	0.239	0.507
20:3(n-6) DGLA	1.74 × 10^−4^	0.009	0.984	0.002	0.010	0.802	0.001	0.007	0.855	0.862
20:4(n-6) AA	−0.004	0.005	0.396	0.003	0.005	0.636	−0.001	0.004	0.774	0.353

*ALA* α-Linolenic acid; *EPA* Eicosapentaenoic acid; *DHA* Docosahexaenoic acid; *LA* Linoleic acid; *GLA* γ-Linolenic acid; *DGLA* Dihomo-γ-linolenic acid; *AA* Arachidonic acid; *MAF* Minor allele frequency. Q_p-value_ Cochran’s Q heterogeneity measure

Beta showed the effect of each percentage change on the change of telomere length (T/S ratio) for n-6 fatty acid, LA and AA, and 1-SD change in the fatty acid in the change of telomere length (T/S ratio) for n6:n3 ratio, n-3 fatty acid, ALA, EPA, DHA GLA and DGLA

### Association between LTL, plasma PUFA and CAD

Higher plasma n6:n3 ratio was significantly associated with increased CAD risk [HR (95%Cl) = 1.114 (1.034, 1.200), *P* = 0.005, (Table 3)]. When analyzing the individual effect of plasma n-3 and n-6 PUFA on LTL, n-3 PUFA showed a significant protective effect on CAD risk [HR (95%Cl) = 0.885 (0.820, 0.955), *P* = 0.002] but not n-6 PUFA (Table 3). We further analyzed the association between specific n-3/n-6 PUFA and CAD risk. Both EPA [HR (95%Cl) = 0.884 (0.820, 0.953), *P* = 0.001] and DHA [HR (95%Cl) = 0.884 (0.819, 0.954), *P* = 0.002] showed significant association with decreased CAD risk (Table 3). The n-6 PUFA subtypes were not associated with CAD (Table 3). We also observed that longer LTL has protective effect on CAD risk [HR (95%Cl) = 0.664 (0.481, 0.917), *P* = 0.013, (Table 3)]. Additionally, we evaluated if the effects of PUFA on CAD was mediated by LTL but did not find strong evidence for this in our dataset (Supplemental Table 4).

**Table 3:** Association between LTL/plasma PUFA and CAD

	HR (95% Cl)	*P*
LTL	0.664 (0.481, 0.917)	**0.013**
n6:n3 ratio	1.114 (1.034, 1.200)	**0.005**
n-3 fatty acid	0.885 (0.820, 0.955)	**0.002**
18:3 (n-3) ALA	0.969 (0.899, 1.044)	0.409
20:5 (n-3) EPA	0.884 (0.820, 0.953)	**0.001**
22:6 (n-3) DHA	0.884 (0.819, 0.954)	**0.002**
n-6 fatty acid	0.998 (0.982, 1.014)	0.798
18:2(n-6) LA	0.999 (0.983, 1.016)	0.940
18:3(n-6) GLA	1.048 (0.969, 1.132)	0.239
20:3(n-6) DGLA	1.038 (0.959, 1.123)	0.354
20:4(n-6) AA	0.980 (0.939, 1.023)	0.366

*ALA* α-Linolenic acid; *EPA* Eicosapentaenoic acid; *DHA* Docosahexaenoic acid; *LA* Linoleic acid; *GLA* γ-Linolenic acid; *DGLA* Dihomo-γ-linolenic acid; *AA* Arachidonic acid; *LTL* Leukocyte telomere length; *HR* Hazard ratio; *Cl* Confidence interval

### Interaction between genetic variants and plasma PUFA on LTL

**Fig. 1:**
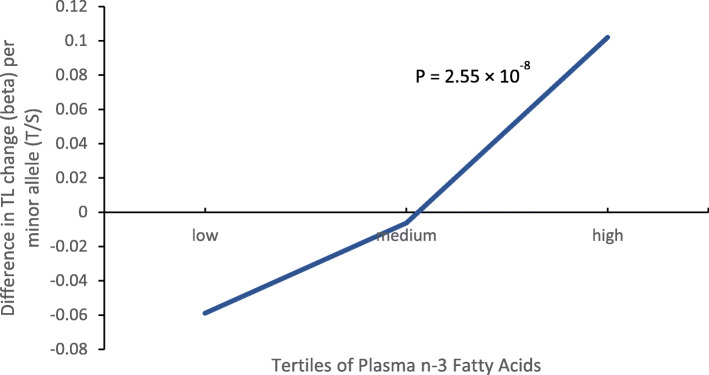
The change of LTL by rs529143 within each tertile of plasma n-3 fatty acid level

**Fig. 2:**
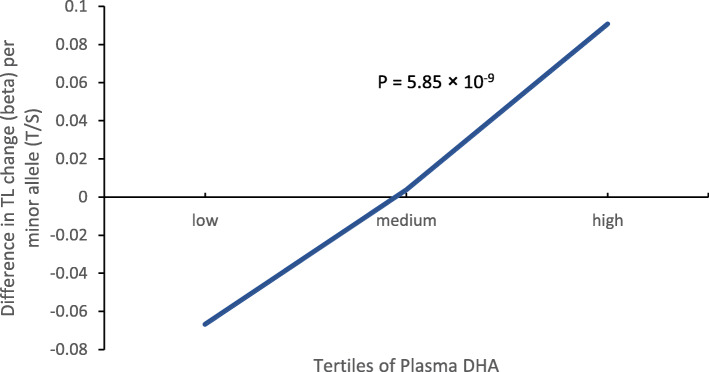
The change of LTL by rs529143 within each tertile of plasma DHA level

In the assessment of the interaction between plasma PUFAs and genetic variants on LTL, an intergenic SNP, rs529143, was found to modify the effect of plasma n-3 PUFA and DHA on LTL both in CAD cases and controls. After meta-analysis, the interaction reached genome-wide level of significance (Table 4, Supplemental Table 1). Although the main effect of rs529143 on LTL was not significant (*p* = 0.252, Supplemental Table 2), interaction existed between rs529143 and n-3 PUFA on LTL. Stratification by tertiles of plasma n-3 PUFA levels indicated that individuals carrying the minor C allele have shorter LTL in the lower tertile group while the higher tertile group have longer LTL (Fig. 1). Similar results were observed for the interaction between DHA and rs529143. Minor CC homozygous subjects have shorter LTL in lower plasma DHA tertile group and longer LTL in higher tertile group (Fig. 2).

**Table 4:** Interaction between genetic variants and plasma PUFA on telomere length

					*N* = 1349			
snp-id	chromosome	position	EA	EAF	beta	se	*p*	Q_p-value_
rs529143 × n-3 fatty acid	1	20,452,020	C	0.131	0.075	0.013	**2.55 × 10**^**−8**^	0.265
rs529143 × DHA					0.078	0.013	**5.85 × 10**^**−9**^	0.223

Q_p-value_ Cochran’s Q heterogeneity measure

*EA* Effect allele; *EAF* Effect allele frequency; *DHA* Docosahexaenoic acid

We further tested whether rs529143 interacted with dietary intake of PUFAs to affect LTL in all the extended SCHS dataset with complete information for both genotype and diet (*N* = 21,828) but no significant interaction was detected (Supplemental Table 3).

## Discussion

In this prospective nested case-control study of the Singaporean Chinese, we observed an inverse association of plasma n6:n3 ratio with LTL and CAD risk. The association was driven by total plasma n-3 but not n-6 PUFA. When studying the association between specific n-3 PUFA and LTL, higher plasma levels of both EPA and DHA were associated with longer LTL and decreased CAD risk. However, the effects of PUFA and LTL on CAD risks were independent in our study population. We further found a genome-wide interaction between an intergenic variant, rs529143, and n-3 PUFA as well as DHA on LTL. To the best of our knowledge, our study represents the first investigation on the effect of plasma PUFA on LTL and its interaction with genetic variants in a Chinese population.

Studies of the association of PUFA, either dietary or in the plasma, with LTL have largely shown inconsistent results. Most have found telomeric attrition to be attenuated by higher plasma n-3 PUFA levels or increased marine n-3 intake [28, 29], which is consistent with the finding that plasma n-3 PUFA concentration is associated with low proinflammatory markers and high anti-inflammatory markers [30]. In contrast, a large cross-sectional study, comprising the controls of the Nurses’ Health Study found no association between n-3 PUFA and LTL. Instead, the study reported increased n-6 PUFA intake, specifically LA intake, to be inversely associated with [31]. In our study, LTL was significantly associated with plasma n-3 but not n-6 levels. One possible explanation for such discrepancies may be due to the varied dietary intakes (and possibly other lifestyle or environmental factors) between the study populations that may impact on LTL attrition rates. Additionally the ratio of plasma n6:n3 levels has not been evaluated extensively in these previous studies for LTL associations. In a randomized controlled trial, there was no significant differences for LTL changes among groups receiving different n-3 PUFA supplementation. However, an increase of LTL was observed with decreasing of n6:n3 ratio [10]. N-3 and n-6 PUFAs compete for key enzymatic pathways, and thus the relative balance is of health interest [16]. Higher plasma n6:n3 ratio has been associated with higher inflammatory markers, such as TNF-α and IL-6 [32]. Oxidative stress, and inflammation may result in LTL attrition [33]. These data, together with the finding in our study, suggest that rather than just considering the absolute amount of n-3 or n-6 PUFA individually, the background n6:n3 ratio should also be taken into account for clinical studies or for evaluation of nutritional interventions. When we tested the association between specific n-3 FAs and LTL, we observed significant association for EPA and DHA but not ALA. Although ALA can be converted to EPA and DHA, the conversion process is inefficient in humans. A previous study had shown that the same dosages of ALA produced different physiological responses from EPA and DHA to decrease risk factors for metabolic syndrome, while physiological responses to EPA and DHA were similar. This result strongly suggests that ALA exerts its independent effects in metabolic syndrome [34]. A randomized double-blind nutritional intervention study also showed that ALA have different effect on cardiovascular risk markers in healthy elderly subjects compared to EPA and DHA [35].

Previous studies have shown an inverse association between long-chain n-3 PUFAs and CAD risk [36] while adipose tissue AA, a n-6 PUFA, was associated with higher risk of AMI [37, 38]. Similar findings in SCHS between plasma PUFA and CAD has been reported previously [20]. In this SCHS data subset with genetic information, higher plasma n6:n3 ratio was associated with shorter LTL and increased CAD risk. The association was driven mainly by elevated total plasma n-3 but not n-6 PUFA, especially EPA and DHA. Since PUFA and TL are both related to oxidative stress and inflammation, which contribute significantly to the pathogenesis of CAD, we investigated whether the effect of PUFA on CAD is mediated through LTL. However, we did find sufficiently strong evidence for this (Supplemental Table 4) and it may be likely that PUFA and LTL have independent effects on CAD risks.

Our interaction analysis indicated an intergenic SNP, rs529143, could modify the association between n-3 PUFA/DHA and LTL. Carriers of the minor C allele with low n-3 PUFA/DHA (lower tertile) had shorter LTL while those with high n-3 PUFA/DHA (higher tertile) had longer LTL. Regional genes (100 kb) around rs529143 include multiple phospholipase genes such as *PLA2G2D* and *PLA2G2F,* which have strong relevance to phospholipid metabolism [39]. Functional annotation of this SNP with expression quantitative trait loci (eQTL) data indicated that rs529143 may affect the expression level of *AKR7A3* (*p* = 4.57 × 10^− 6^) in transformed fibroblasts [40, 41], which is involved in the detoxification of aldehydes and ketones. The enzymes from the aldo-keto reductases (AKRs) superfamily were also reported to play important roles in nuclear receptor signaling, cellular metabolism, inflammatory responses, endobiotic, osmoregulation and xenobiotic detoxification and hormone synthesis [42, 43]. Moreover, genome-wide yellow fluorescent protein complementation screen has showed that AKR7A3 can interact with DNA-binding transcription factor Ras-related protein 1 (RAP1), one of the core telomeric proteins, to regulate telomeres [44]. The interaction observed in our study might be through the effect of AKR7A3 on telomere length.

Our study has several potential limitations. First, measurements of LTL in our study were mean TL in leukocytes and therefore may not reflect TL dynamics in other tissues [45]. The measurements of TL in vascular cells could be more informative for the mediation analysis for CAD effects [46]. However, there is evidence that within an individual, LTL is likely to be correlated with tissue specific TL [47, 48]. Second, although the association between plasma PUFA levels and LTL was significant in the meta-analysis, when examining the association in cases and controls separately, they were only significant in the latter. Nevertheless the direction of the association was consistent across the datasets and the between-study heterogeneity examined by Cochran’s Q test was not significant (*P* > 0.05).

## Conclusions

We report in this study an inverse association of plasma n6:n3 ratio with LTL and CAD risk and that this association was mainly driven by total plasma n-3 but not n-6 PUFA. Higher plasma levels of both EPA and DHA were associated with longer LTL and decreased CAD risk. We additionally identified an intergenic genetic variant, rs529143 that was observed to modify the association between plasma n-3 PUFA/DHA level and LTL.

## Supplementary information

**Additional file 1: Table S1.** Interaction between genetic variants and plasma PUFA on telomeres in SCHS_CAD cases and controls. **Table S2.** Association between genetic variant and telomeres. **Table S3.** The mediation effect of telomeres on the association between plasma PUFA and coronary artery disease. **Table S4.** Interaction between genetic variants and PUFA intake on telomeres in SCHS (*N* = 21,828)

## Data Availability

All data analyzed during this study are included in this published article and its supplementary information files.

## Abbreviations

ALA: α-Linolenic acid
GLA: γ-Linolenic acid
AMI: Acute myocardial infarction
AKR: Aldo-keto reductase
AA: Arachidonic acid
CAD: Coronary artery disease
DGLA: Dihomo-γ-linolenic acid
DHA: Docosahexaenoic acid
EPA: Eicosapentaenoic acid
eQTL: Expression quantitative trait loci
GC-MS: Gas chromatography–mass spectrometry
HWE: Hardy–weinberg expectations
LTL: Leukocyte telomere length
LA: Linoleic acid
MS: Mass spectrometry
MAF: Minor allele frequency
n6:n3 ratio: N-6 and n-3 PUFA
n-3: Omega 3
n-6: Omega-6
PUFA: Polyunsaturated fatty acid
PC: Principle components
QC: Quality control
qPCR: Quantitative PCR
RAP1: Ras-related protein 1
SCHS: Singapore Chinese health study
SNP: Single nucleotide polymorphism
SD: Standard deviation
TL: Telomere length
