# Supplementary material for: Effect of plasma polyunsaturated fatty acid levels on leukocyte telomere lengths in the Singaporean Chinese population
Source: Nutr J. 2020 Oct 30;19:119. doi: 10.1186/s12937-020-00626-9 (PMC7602302; doi:10.1186/s12937-020-00626-9)
Supplement: Supplementary file 1 — Additional file 1: Table S1. Interaction between genetic variants and plasma PUFA on telomeres in SCHS_CAD cases and controls. Table S2. Association between genetic variant and telomeres. Table S3. The mediation effect of telomeres on the association between plasma PUFA and coronary artery disease. Table S4. Interaction between genetic variants and PUFA intake on telomeres in SCHS (N = 21,828) [file 12937_2020_626_MOESM1_ESM.docx]

**S1 Table.** Interaction between genetic variants and plasma PUFA on telomeres in SCHS_CAD cases and controls

|  |  |  |  | |  | SCHS_case | | | SCHS_control | | |
| --- | --- | --- | --- | --- | --- | --- | --- | --- | --- | --- | --- |
| snp-id | chromosome | position | | EA | EAF | beta | se | p | beta | se | p |
| rs529143 × n-3 fatty acid | 1 | 20452020 | | C | 0.131 | 0.063 | 0.017 | **3.19 × 10^-4^** | 0.094 | 0.021 | **1.11 × 10^-5^** |
| rs529143 × DHA |  |  |  |  |  | 0.064 | 0.017 | **1.77 × 10^-4^** | 0.098 | 0.021 | **3.91 × 10^-6^** |

EA: Effect allele; EAF: Effect allele frequency; EPA: Eicosapentaenoic acid; DHA: Docosahexaenoic acid.

**S2 Table.** Association between genetic variant and telomeres.

|  | SCHS_case | | | SCHS_control | | | Meta-analysis | | | |
| --- | --- | --- | --- | --- | --- | --- | --- | --- | --- | --- |
|  | N = 711 | | | N = 638 | | | N = 1349 | | | |
|  | beta | se | p | beta | se | p | beta | se | p | Q_p-value_ |
| rs529143 | 0.017 | 0.019 | 0.371 | 0.015 | 0.020 | 0.473 | 0.016 | 0.014 | 0.252 | 0.943 |

Q_p-value_ Cochran’s Q heterogeneity measure.

**S3 Table.** The mediation effect of telomeres on the association between plasma PUFA and coronary artery disease

|  | total effect | | | indirect effect | | | direct effect | | |
| --- | --- | --- | --- | --- | --- | --- | --- | --- | --- |
|  | beta | se | p | beta | se | p | beta | se | p |
| n6:n3 ratio | 0.030 | 0.014 | 0.029 | 0.001 | 0.001 | 0.209 | 0.028 | 0.014 | 0.037 |
| n-3 fatty acid | -0.036 | 0.014 | 0.008 | -0.001 | 0.001 | 0.218 | -0.035 | 0.014 | 0.011 |
| 18:3 (n-3) ALA | -0.013 | 0.014 | 0.349 | 0.000 | 0.001 | 0.752 | -0.013 | 0.014 | 0.355 |
| 20:5 (n-3) EPA | -0.045 | 0.014 | 0.001 | -0.001 | 0.001 | 0.226 | -0.044 | 0.014 | 0.001 |
| 22:6 (n-3) DHA | -0.034 | 0.014 | 0.011 | -0.001 | 0.001 | 0.214 | -0.033 | 0.014 | 0.015 |
| n-6 fatty acid | -0.004 | 0.003 | 0.215 | 7.25 × 10^-5^ | 1.33 × 10^-4^ | 0.586 | -0.004 | 0.003 | 0.205 |
| 18:2(n-6) LA | -0.003 | 0.003 | 0.322 | 6.15 × 10^-5^ | 1.33 × 10^-4^ | 0.644 | -0.003 | 0.003 | 0.312 |
| 18:3(n-6) GLA | 0.009 | 0.014 | 0.522 | -6.90 × 10^-4^ | 7.52 × 10^-4^ | 0.356 | 0.010 | 0.014 | 0.490 |
| 20:3(n-6) DGLA | 0.007 | 0.014 | 0.634 | -4.20 × 10^-5^ | 6.08 × 10^-4^ | 0.945 | 0.007 | 0.014 | 0.632 |
| 20:4(n-6) AA | -0.008 | 0.008 | 0.306 | 8.18 × 10^-5^ | 3.42 × 10^-4^ | 0.811 | -0.008 | 0.008 | 0.301 |

ALA: α-Linolenic acid; EPA: Eicosapentaenoic acid; DHA: Docosahexaenoic acid.

**S4 Table.** Interaction between genetic variants and PUFA intake on telomeres in SCHS (N = 21828)

|  | rs529143 | | | | | |
| --- | --- | --- | --- | --- | --- | --- |
|  | non EA | EA | EAF | beta | se | p |
| Omega 3 fatty acid | A | C | 0.177 | 0.154 | 0.266 | 0.563 |
| Omega 3 fatty acid from fish |  |  |  | -0.073 | 0.170 | 0.668 |
| Omega 3 fatty acid from others |  |  |  | 0.100 | 0.105 | 0.344 |
| Omega 6 fatty acid |  |  |  | 0.006 | 0.009 | 0.516 |

EA: Effect allele; EAF: Effect allele frequency.
